# DL-PPI: a method on prediction of sequenced protein–protein interaction based on deep learning

**DOI:** 10.1186/s12859-023-05594-5

**Published:** 2023-12-14

**Authors:** Jiahui Wu, Bo Liu, Jidong Zhang, Zhihan Wang, Jianqiang Li

**Affiliations:** 1 Faculty of Information Technology, Beijing University of Technology, Beijing, 100124 China; 2 School of Mathematical and Computational Sciences, Massey University, Auckland, 0745 New Zealand

**Keywords:** Protein–protein interaction, Deep learning, Graph neural network, Feature extraction

## Abstract

**Purpose:**

Sequenced Protein–Protein Interaction (PPI) prediction represents a pivotal area of study in biology, playing a crucial role in elucidating the mechanistic underpinnings of diseases and facilitating the design of novel therapeutic interventions. Conventional methods for extracting features through experimental processes have proven to be both costly and exceedingly complex. In light of these challenges, the scientific community has turned to computational approaches, particularly those grounded in deep learning methodologies. Despite the progress achieved by current deep learning technologies, their effectiveness diminishes when applied to larger, unfamiliar datasets.

**Results:**

In this study, the paper introduces a novel deep learning framework, termed DL-PPI, for predicting PPIs based on sequence data. The proposed framework comprises two key components aimed at improving the accuracy of feature extraction from individual protein sequences and capturing relationships between proteins in unfamiliar datasets. 1. Protein Node Feature Extraction Module: To enhance the accuracy of feature extraction from individual protein sequences and facilitate the understanding of relationships between proteins in unknown datasets, the paper devised a novel protein node feature extraction module utilizing the Inception method. This module efficiently captures relevant patterns and representations within protein sequences, enabling more informative feature extraction. 2. Feature-Relational Reasoning Network (FRN): In the Global Feature Extraction module of our model, the paper developed a novel FRN that leveraged Graph Neural Networks to determine interactions between pairs of input proteins. The FRN effectively captures the underlying relational information between proteins, contributing to improved PPI predictions. DL-PPI framework demonstrates state-of-the-art performance in the realm of sequence-based PPI prediction.

**Supplementary Information:**

The online version contains supplementary material available at 10.1186/s12859-023-05594-5.

## Introduction

Proteins constitute a central focus of investigation across multiple research domains, given their critical role as the primary organic components of cells. Rather than functioning in isolation, proteins engage in intricate interactions, catalyzing reactions between multiple proteins to accomplish specific tasks [1]. These interactions, known as Protein–Protein Interactions (PPIs), manifest as physical contacts between two or more proteins. Leveraging PPIs holds tremendous potential in diverse life science fields, such as medical diagnosis, drug development, and disease treatment [2].

The prediction of protein interaction relationships offers valuable insights into shared functionalities and processes among different types of cancers [3], as well as the underlying pathogenic mechanisms of inherited neurodegenerative diseases in humans [4]. Moreover, it facilitates the construction of protein interaction networks [5]. However, traditional biological methods for predicting protein interactions, such as yeast two-hybrid screens [6], tandem affinity purification [7], and protein chips [8], have encountered challenges, including the generation of false positives due to promiscuous proteins and issues related to high costs and labor-intensive procedures. The wealth of information contained in PPI data necessitates the development of novel computational tools to enable transformative biological discoveries. To this end, there is a pressing need for more efficient and computer-dependent algorithms that streamline the prediction of protein interactions while minimizing labor requirements.

Among the protein interaction prediction methodologies in the domain of computer science, three principal categories are distinguished based on the biological information of proteins: structure-based models [9–11], gene-ontology-based models [12, 13], and sequence-based models [14–16]. The first approach primarily revolves around the development of prediction models that rely on the frequency characteristics of interactions observed between pairs of structural domains. Nevertheless, the predictive accuracy of such models is impeded by the limited availability of samples. In contrast, gene-ontology-based models harness the semantic similarity derived from Gene Ontology (GO) annotations, which has emerged as one of the most potent indicators of protein interactions [17]. Lastly, the third method, sequence-based models, takes precedence over other strategies owing to their independence from specific information about protein properties, allowing them to perform their predictive tasks based solely on the protein sequences [18–20]. These sequence-based methods leverage the inherent information encoded in protein sequences to infer interaction patterns, thus offering a versatile and data-driven approach to protein interaction prediction. In conclusion, each category of prediction methods presents distinct merits and limitations, and the selection of an appropriate approach is contingent upon the specific research objectives and the availability of data resources.

These approaches rely on several feature extraction processes for the protein sequences. In the research conducted by Shen et al. [21], this method harnessed the power of a support vector machine, combining it with a kernel function and a conjoint triad feature to achieve an impressive average accuracy of 83.90 ± 1.29%. Each training set consisted of 32,486 protein pairs; half of the protein pairs were randomly selected from the data of positive PPI pairs, and the other half were randomly selected from the negative protein pairs. Model PCA-EELM [22], the research present a novel model to predict PPI only using the information of protein sequences. In the proposed method, 11,188 protein pairs retrieved from the DIP database were encoded into feature vectors by using four kinds of protein sequences information. We can see that the model gives good prediction performance with an average Sens. value of 86.15%, Prec. value of 87.59% and MCC value of 77.36%. In the research conducted by Sun et al. [23], this research is the first to apply a deep-learning algorithm to sequence-based PPI prediction. The prediction accuracies for various external datasets ranged from 87.99 to 99.21%, which are superior to those achieved with previous methods. Model DeepPPI [24] employs deep neural networks to learn effectively the representations of proteins from common protein descriptors. DeepPPI harnessed deep neural networks to predict PPIs effectively, delivering an exceptional performance with an Accuracy of 92.50%, Precision of 94.38%, Recall of 90.56%, Specificity of 94.49%. These features measure physicochemical properties of the 20canonical amino acids, and aim at summarizing full sequence information relevant to PPIs.

The application of computer methods for predicting Protein–Protein Interactions (PPI) can be divided into two main stages. The initial phase was dominated by Machine Learning technologies [25], involving the construction of linear relationships and training classifiers [26]; including Weighted Sparse Representation-based Classifier [27], SVM (Support Vector Machine) [28–30], Random Forest [31], Rotation Forest [32], KNN (K-Nearest Neighbors) [33], Extreme Learning Machine (ELM) [34], and other Support Vector Machines [35].

In recent years, Deep Learning has emerged as a promising technology capable of learning protein features more accurately and automatically, thereby enhancing the accuracy of PPI prediction. Numerous studies have employed Convolutional Neural Networks (CNN) to extract features from protein amino acid sequences [36–40]. In 2016, an article proposed a Graph Convolutional Neural Network (GCN) model that incorporates graph-structured data into CNN to improve feature extraction accuracy [41]. Subsequently, the latest research has demonstrated the application of Graph Convolutional Networks (GCNs) in protein interaction prediction and classification tasks [42]. GCNs function as feature extractors similar to CNNs, employing these features for node classification, graph classification, link prediction, and embedding representation of graph structures. Notably, GCNs exhibit powerful capabilities in aggregating information from neighboring nodes in graph-structured data.

However, it is important to acknowledge that GCN is still in the early stages of development, and many shallow models may struggle to capture features from higher-order neighbors. Additionally, some GCN models have shown limited effectiveness when applied to unknown PPI datasets [43]. Further advancements in GCN methodologies are necessary to address these challenges and enhance the robustness of PPI prediction in diverse and unfamiliar protein interaction datasets.

Since the introduction of AlexNet [44], Convolutional Neural Networks (CNNs) have made remarkable strides by increasing network depth and width while reducing parameters. However, this advancement has led to the issue of gradient vanishing, which hampers training. To address this concern, the Inception model [45] was proposed, incorporating the innovative $1\times 1$ convolution kernel from the Network in Network (NIN) architecture [46]. This $1\times 1$ convolution adds a lightweight layer of feature transformations, deepening the neural network while maintaining computational efficiency. Additionally, the introduction of Batch Normalization normalizes the input value distribution for each layer, ensuring that it falls into the sensitive region of the activation function and mitigates the problem of vanishing gradients. A significant contribution of the Inception model lies in the utilization of the Inception module, which employs $1\times 1$ convolution kernels in each branch.

In the subsequent paper, Inception V3 [47], the concept of factorial decomposition is introduced to enhance the performance of Inception V1. The underlying principle is to achieve a balance between learning rich feature representations and reducing computational complexity to avoid bottlenecks in feature representation throughout the network. The approach involves substituting large convolution filters with a multilayer perceptron (MLP) and decomposing convolutions to reduce computation. Specifically, an $n\times n$ convolution can be replaced by $1\times n$ and $n\times 1$ convolutions, leading to more efficient and effective feature learning.

The Attention Mechanism is a technique that involves assigning weights to input elements to highlight the importance of certain features while de-emphasizing others, ultimately generating a weighted sum for a specific target. This mechanism is particularly useful when dealing with sets of input vectors and output vectors, both of varying lengths (N). By applying the self-attention mechanism, the problem of establishing correlations for multiple correlated inputs, which cannot be fully addressed by traditional fully connected neural networks, can be effectively resolved. The self-attention mechanism enables the model to recognize the interconnections between different components within the entire input.

The Attention Mechanism initially emerged in the 1990s, finding applications in computer vision. In 2014, Google Mnih et al. [48] integrated the Attention Mechanism into Recurrent Neural Networks (RNN) for image classification, achieving impressive results. Subsequently, the Attention Mechanism gained widespread attention in the field of deep learning and natural language processing. In particular, Bahdanau et al. [49] incorporated the attention mechanism into an encoder-decoder framework for translation tasks, yielding favorable outcomes. However, the real breakthrough for the attention mechanism occurred in 2017 when Google introduced the Transformer model [50]. The Transformer introduced the concept of Self-Attention Mechanism, departing from traditional RNN and CNN architectures, and fully exploiting the capabilities of Deep Neural Networks (DNN). This revolutionary development significantly impacted the deep learning field, elevating the prominence of the attention mechanism.

This research paper introduces DL-PPI: Graph Neural Network for sequence-based Protein–Protein Interaction (PPI) Prediction, which presents an end-to-end deep learning framework tailored for sequence-based PPI prediction tasks. In the approach, proteins are treated as nodes, and protein interactions are represented as edges, resulting in the construction of undirected graphs. PPI prediction is framed as a link prediction problem, wherein the matrix X is formed using the features of each protein node, and the relationship between each node (PPI) is used to construct the adjacency matrix A, serving as the input to our model. By processing the graph with DL-PPI and leveraging Graph Neural Networks (GNNs), the paper aims to extract relationship features between proteins and consequently infer interactions involving unknown proteins.

In summary, this paper contributes to three main aspects. Firstly, the paper proposes an end-to-end graph neural network-based model, specifically designed for predicting Protein–Protein Interactions between novel proteins. This model takes the sequence features of two proteins as input and predicts whether an interaction will occur. Secondly, we introduce a novel graph similarity algorithm that enhances the interaction representation between two protein node features, leading to more accurate relationship classification in the prediction module. Lastly, the proposed DL-PPI model demonstrates superior performance across different datasets, outperforming the GNN-PPI baseline [43] and proving to be more effective in predicting Protein–Protein Interactions.

## Methods

### Dataset

*STRING* STRING version 10.5 [51] is a publicly available dataset, widely utilized in prior research. This dataset systematically integrates both known and predicted Protein–Protein Interactions (PPIs), encompassing direct (physical) interactions, as well as indirect (functional) interactions. The dataset includes interactions of seven types: activation, binding, catalysis, expression, inhibition, post-translational modification, and reaction.

For the PPI prediction task, the paper adopted two datasets utilized in the PIPR study [52]. The first dataset, SHS148k, comprises 44,488 multi-label PPIs, while the second dataset, SHS27k, consists of 7624 multi-label PPIs. Both datasets were randomly extracted from the Homo sapiens (Homo sapiens is a subset of the STRING database, all data related to Homo sapiens of organisms), ensuring that they share less than 40% of sequence identity.

Furthermore, the entire protein sequence from the Homo sapiens subset of STRING was utilized as the third dataset, namely STRING, which encompasses a total of 593,397 PPIs [43].

*Yeast dataset* The Yeast PPI dataset is a widely recognized benchmark dataset extensively utilized in state-of-the-art methods [22, 29, 32, 53]. It comprises 2497 proteins, resulting in a total of 11,188 PPIs, evenly split between positive and negative cases. The positive cases were derived from the DIP_20070219 database of interacting proteins [54]. The model incorporates full protein sequences sourced from UniProt [55]. Negative interactions were generated by randomly pairing proteins without any documented evidence of interaction.

These datasets were used to perform the Protein–Protein Interaction prediction task in the study.

### Architecture

﻿This paper ﻿introduces DL-PPI, a comprehensive end-to-end deep learning framework specifically tailored for addressing PPI prediction tasks. In this context, the PPI prediction problem is formulated as a multivariate classification task with seven distinct classes, all based on protein sequence data. DL-PPI is designed to capitalize on extensive protein and PPI datasets during the training process, empowering it to effectively predict interactions between two input proteins and ascertain the corresponding types of interactions based on the inherent sequence characteristics of the proteins. The DL-PPI method involves four main steps:

1. *Data Pre-processing* PPI data is pre-processed using a pre-trained embedding model to encode the protein information effectively.
2. *Protein Node Feature Extraction* This step focuses on capturing protein features encoded in a one-dimensional space through sequence feature learning. Each protein undergoes processing using Inception methods to derive protein node-level features.
3. *Global Feature Extraction* Protein maps are constructed, and a Graph Neural Network (GNN) is employed to learn the topology and relationships between proteins at a global level.
4. *PPI Prediction* The features obtained in the previous step are input into a self-attention mechanism module, resulting in two protein features enriched with essential information. Subsequently, the interaction features of the two nodes are fused, and the type of interaction relationship between them is predicted using a Feature Relational Reasoning Network (FRN).

**Fig. 1:**
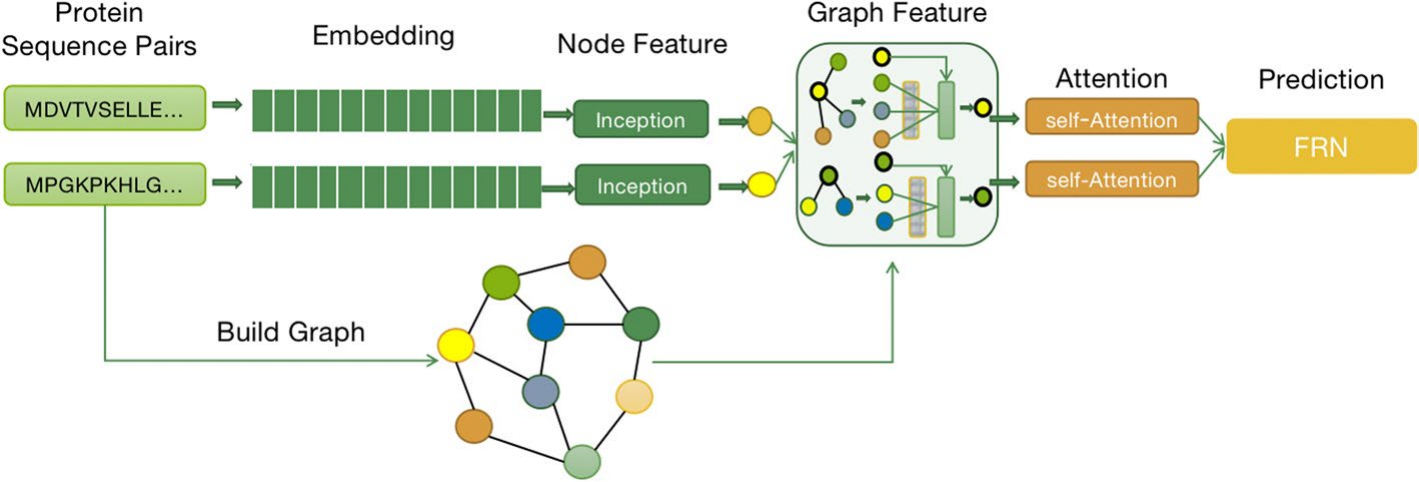
The network architecture of the proposed DL-PPI model

The overall architecture of the DL-PPI learning process is illustrated in Fig. 1. This comprehensive approach enables DL-PPI to effectively learn from vast protein and PPI datasets, leading to accurate predictions of protein interactions and their corresponding interaction types.

### Data pre-processing

The use of amino acid embedding enables the transformation of proteins into distinct dimensional spaces, allowing for the extraction of multidimensional information about these proteins. By representing proteins in these embedded spaces, the model can capture intricate relationships and patterns among amino acids, ultimately encoding a comprehensive representation of the protein’s characteristics.

Amino acid embedding leverages techniques inspired by word embedding in natural language processing, where words are mapped to continuous vectors in a lower-dimensional space. Similarly, in protein sequences, each amino acid is represented by a unique vector in the embedded space, preserving relevant information while reducing the dimensionality of the data. This process enhances the model’s ability to recognize similarities, correlations, and functional characteristics among different proteins based on their embedded representations.

*Amino acid embeddings*

In our approach, each amino acid vector, denoted as *a*, is represented as the sum of two subembeddings, i.e., $a = {a_1, a_2}$. The first part, $a_1$, captures the co-occurrence similarity of amino acids and is obtained by pre-training the Skip-Gram model [56] on protein sequences. The skip-gram model is trained using negative sampling, where the vocabulary samples are overlapping 3-mer amino acids, and the word vector size is 5. This setup has achieved good performance on phrase datasets and allows them to quickly compare negative sampling and hierarchical Softmax with or without quadratically sampling high-frequency tokens.

The second part, $a_2$, reflects the categorization of electrostaticity and hydrophobicity of amino acids and is represented as a seven-dimensional vector. The 20 natural amino acids are grouped into 7 classes based on their properties [21]. Additionally, the 21st amino acid, U (Selenocysteine), and the 22nd amino acid, O (Pyrrolysine), along with other unknown proteins, are grouped into an eighth class. Consequently, each amino acid vector is represented as 13 dimensions (5 + 7 + 1) [43].

By employing this dual subembedding representation, it is effective to capture both co-occurrence similarity and categorization information of amino acids, facilitating a comprehensive and informative representation of proteins in a lower-dimensional space. This enhanced representation is crucial for improving the performance of the DL-PPI framework in predicting Protein–Protein Interactions and gaining valuable insights into the complex interactions among proteins.

### Protein node feature extraction

In previous studies [43, 52], it has been demonstrated that feature extraction from amino acid sequences in proteins can significantly enhance the accuracy of PPI prediction. To this end, the paper employs the Inception V3 model for protein nodes based on amino acid sequences.

*Inception module*

**Fig. 2:**
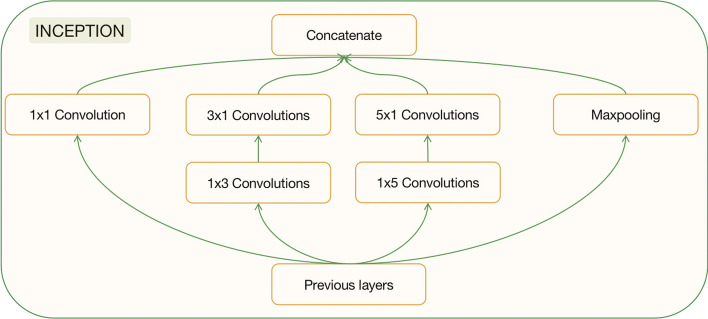
Inception architecture. Concatenated together with convolution kernels of 1, 3, and 5, respectively, and maxpooling

In the context of the Inception module, the matrix dimension is denoted as $X \in R^{a \times L}$, where *a* represents the feature dimension (set as 13) obtained from the previous step. Inception V3 is utilized to capture the distinctive features of the protein sequence. This model comprises four sub-modules, structured as depicted in Fig. 2. The primary principle underlying this model involves employing convolution kernels of different sizes, namely 1, 3, and 5, which implies the use of varying receptive fields. Subsequently, the outputs of these convolutional layers are concatenated together to fuse features at different scales, enhancing the network’s nonlinearity and enabling the extraction of diverse and informative features.

By integrating the Inception module into the DL-PPI framework, this paper aims to leverage the power of multi-scale feature extraction to better capture the intricate characteristics of protein sequences and improve the accuracy of PPI prediction. This approach enhances the model’s ability to discern subtle patterns and correlations between amino acid sequences, thus facilitating more precise and reliable predictions of protein interactions.

### Global feature extraction

In this section, the researchers explore the methodologies employed in previous studies [43] for learning graph-level features. To construct the PPI network, denoted as $G = (P, X)$, this paper utilizes the training dataset, where *P* represents the proteins and *X* represents the interactions between proteins.

The protein network graph is constructed utilizing all the training sets. For enhanced generalization capability and improved performance in predicting unknown proteins, it is advisable not to include all proteins. This is especially relevant since, for unknown proteins, their neighboring nodes are typically unknown. The protein node features obtained from the protein node feature extraction module are employed as input for the protein graph. Within this graph, protein data serve as nodes, protein interaction data function as edges, and the graph is systematically built by identifying and amalgamating common protein interaction types among different proteins as links to the edges. This approach ensures a more robust representation of the protein network, enhancing its utility in predicting interactions involving previously uncharacterized proteins.

Through this construction process, the model demonstrates robustness in predicting interactions involving unknown proteins. Using the Graph Isomorphic Network (GIN) approach [57] for learning graph-level features, it utilizes the sum of neighboring nodes’ features as the aggregation function, while employing multilayer perceptrons (MLPs) to update the aggregated functions. The update of node features in the GIN model is represented by Eq. 1:

1 $$\begin{aligned} g^k_p = MLP^k\left( (1+\epsilon ^k) \cdot g^{k-1}_p + \sum \limits _{{p^\prime \in N(p)}}g^{k-1}_{p\prime } \right) \end{aligned}$$

where $g^k_p$ denotes the feature of node *p* at the *k*th iteration. *k* represents the iterations, $\epsilon$ is a learnable parameter for each layer.

By leveraging the GIN model to update node features within the PPI network, the model can effectively learn comprehensive graph-level representations that encapsulate the complex relationships and interactions between proteins. This approach empowers the DL-PPI framework to achieve improved predictive performance, particularly when dealing with interactions involving previously unseen proteins.

### Prediction

#### Self-attention

The Attention mechanism involves processing the input raw data in the form of <Key, Value> pairs. Given a specific Query, the goal is to calculate the similarity coefficient between the Key and Query, resulting in the corresponding weight coefficient for the associated Value. These weight coefficients are then used to weight and sum the Values, yielding the final output.

In the context of the DL-PPI framework, we use *Q*,*K* and *V* to represent the Query, Key, and Value, respectively. The formula for calculating the attention weight factor, denoted as *W*, is shown in Eq. 2:

2 $$\begin{aligned} W = \text {Softmax}(\frac{Qk^T}{\sqrt{d_k}}) \end{aligned}$$

where Softmax is the softmax function, $d_k$ is the dimension of the Key, and $Qk^T$ represents the dot product between the Query and the transpose of the Key.

The self-attention mechanism, represented as Eq. 3, is a crucial component in the DL-PPI framework:

3 $$\begin{aligned} \text {Attention}(Q,K,V) = W \cdot V = \text {softmax}(QK^T) \cdot V \end{aligned}$$

In this mechanism, *Q*,*K*, and *V* are derived from the same matrix by different linear transformations. This design choice enables the self-attention mechanism to focus on the connections within the inputs, thereby extracting more comprehensive feature information from the protein nodes. This attribute makes it particularly suitable for predicting interactions involving unknown proteins.

The self-attention mechanism is mathematically formulated as follows Eq. 4:

4 $$\begin{aligned} \text {Self-Attention}(X) = \text {Attention}(XW_Q, XW_K, XW_V) \end{aligned}$$

where *X* represents the input matrix, and $W_Q$, $W_K$, $W_V$ are learnable weight matrices for the linear transformations.

Indeed, by incorporating the attention mechanism, the DL-PPI framework can effectively concentrate on the most relevant information and features within the data. This allows the model to capture and exploit the intrinsic relationships and dependencies present in the protein nodes, while reducing the emphasis on less important elements. The attention-weighted aggregation process enhances the feature extraction capabilities of our framework and leads to improved prediction accuracy, particularly for interactions involving unknown proteins.

#### FRN (Feature relational reasoning network)

The objective of Neural Tensor Networks (NTN) [58] is to determine whether two entities, denoted as $(e_1, e_2)$, are related through some specific relation *R*. Let $(e_1, e_2) \in \mathcal {R}^d$ represent the vector representations or features of the two entities. In NTN, the standard linear neural network layer is replaced with a bilinear tensor layer that directly links the two entity vectors in multiple dimensions. The key advantage of NTN lies in its ability to relate the two inputs multiplicatively, rather than implicitly through the nonlinearity, as seen in standard neural networks where the entity vectors are simply concatenated.

To calculate a likelihood score for the existence of a relationship between the two entities, the NTN-based function is formulated as Eq. 5:

5 $$\begin{aligned} g(e_1,R,e_2)= u^T_R \cdot f \left( e^T_1 \cdot W^{[1:k]}_R \cdot e_2 +V_R \begin{bmatrix} e_1 \\ e_2 \end{bmatrix}+b_R\right) \end{aligned}$$

where $f=tanh$ represents the standard element-wise nonlinearity applied to the vector. The tensor $W^{[1:k]} \in \mathbb R^{d\times d\times k}$ is a bilinear tensor product that results in a vector$h \in \mathbb R^k$, where each entry is computed by one slice, denoted as $i = 1, \ldots , k$ of the tensor: $h_i = e^T_1 W^{[i]}_R$.

The parameters specific to relation *R* are represented by $V_R \in \mathbb R^{k \times 2d}$ and $U \in \mathbb R^k$, along with $b_R \in \mathbb R^k$ following the standard form of a neural network.

By utilizing NTN, the model can effectively capture complex relationships and interactions between entities in multiple dimensions, enabling more expressive representations and improving the capability of the model to reason about relationships and make accurate predictions. The NTN-based function plays a critical role in enhancing the overall performance of the model in relation prediction tasks.

The NTN has demonstrated superior performance in computing relationship scores compared to traditional methods. However, its reliance solely on parameter updates during training overlooks the internal correlation between pairs of input vectors, potentially affecting the task of fine similarity computation.

**Fig. 3:**
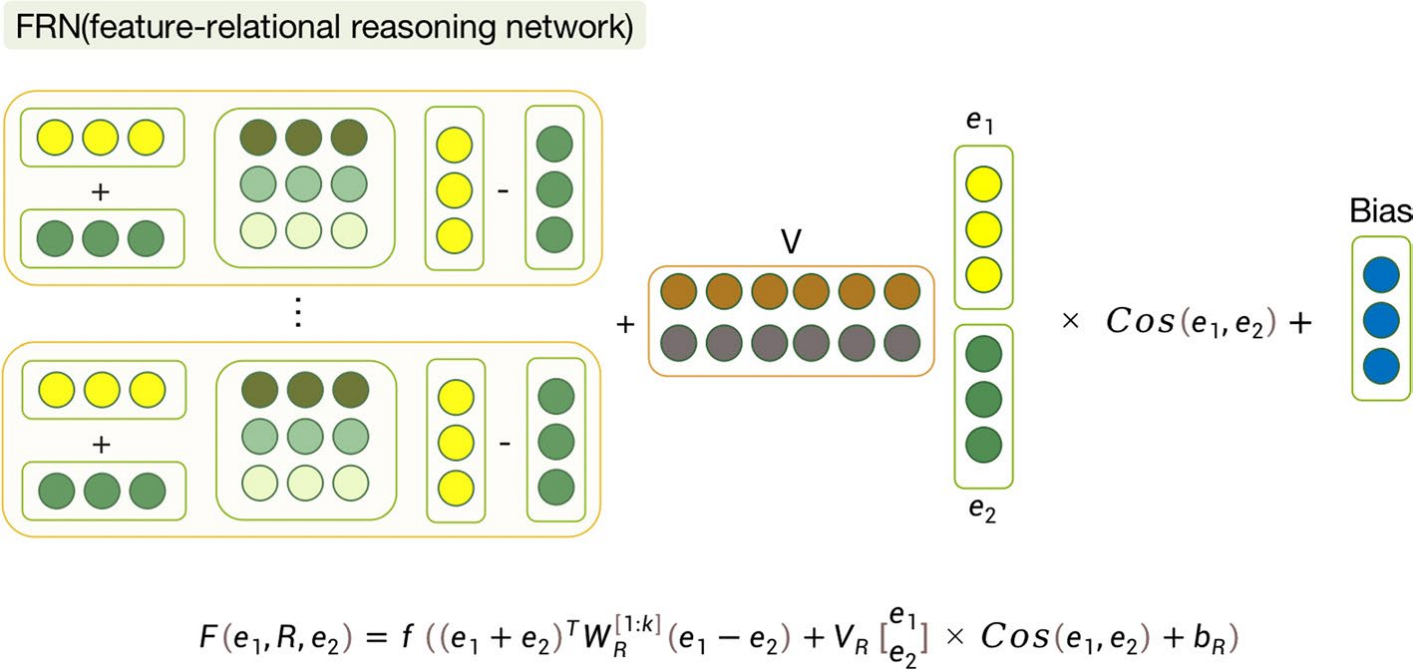
FRN architecture

To address this limitation, this paper proposed a new method called the Feature Relational Reasoning Network (FRN). The structure of the FRN module is illustrated in Fig. 3. In this module, it takes the two protein feature vectors, $e_1$ and $e_2$, obtained from the Attention module in the previous section as inputs. Since $e_1$ and $e_2$ are in the same vector space, the model can easily deduce their distance and direction, which are common similarity metrics for pairs of feature vectors.

In the redesigned FRN module, the paper visualizes the interactions as follows in Eq. 6:

6 $$\begin{aligned} F(e_1, R, e_2) = f\left( (e_1 + e_2)^T \cdot W^{[1:k]}_R \cdot (e_1 - e_2) + V_R \begin{bmatrix} e_1 \\ e_2 \end{bmatrix} \cdot \cos ({e_1,e_2})+b_R\right) \end{aligned}$$

where $F(e_1,R,e_2)$ represents the relation between $e_1$ and $e_2$, $f(\cdot )$ denotes the tanh function. $W^{[1:k]}_R$ is a slice of the tensor, and $b_R$ represents bias term. $$V_R \begin{bmatrix} e_1 \\ e_2 \end{bmatrix}$$ is the standard layer, and $$\begin{bmatrix} e_1 \\ e_2 \end{bmatrix}$$ denotes the concatenation operation of the pairwise inputs $e_1$ and $e_2$.

Additionally, to capture the cosine similarity between $e_1$ and $e_2$, we introduce the term $\cos ({e_1,e_2})$ as Eq. 7:

7 $$\begin{aligned} \cos ({e_1,e_2})=\frac{e_1 \cdot e_2}{\left| e_1 \right| \times \left| e_2 \right| } \end{aligned}$$

Incorporating the FRN module considers both the distance and direction between the protein feature vectors, enabling more refined similarity computation. By integrating this novel approach, the DL-PPI framework gains the capability to make more accurate and informative predictions about protein–protein interactions, particularly for unknown proteins.

#### Multi-label PPI prediction

In the final step of the DL-PPI framework, the model utilizes a fully connected layer (FC) as a classifier for multi-label PPI prediction. The activation function used in this layer is the Rectified Linear Unit (RELU).

For the multi-label PPI prediction task, the paper employs the Multi-task Binary Cross-Entropy Loss ($L_{CE}$) as the loss function for the model, formulated as Eq. 8:

8 $$\begin{aligned} L_{CE} = \sum _{n}^{k=0} \left( \sum _{x_{ij}\in x_{train}} -y^k_{ij} \log {\hat{y}^k_{ij} }-(1-y^k_{ij}\log (1-\hat{y}^k_{ij})) \right) \end{aligned}$$

where $\hat{y}_{ij} = FC(e_1 \cdot e_2)$, $e_1$ and $e_2$ are the protein feature vectors obtained from the Attention module. $y^k_{ij}$ represents the ground truth label for the k-th interaction type between proteins $e_i$ and $e_j$, and $\hat{y}^k_{ij}$ denotes the predicted probability of this interaction type.

The Multi-task Binary Cross-Entropy Loss enables the DL-PPI model to simultaneously predict multiple interaction types between proteins, considering the binary classification nature of each interaction type (either present or not present). By employing this loss function, the model can effectively learn the relationships between proteins and make accurate predictions for various types of interactions. The FC layer with RELU activation function serves as a crucial component in the classification process, contributing to the model’s capability to handle multi-label PPI prediction tasks.

## Results

In this section, the model presents the results of the proposed method on three distinct datasets: SHS27k, SHS148k, and STRING. The paper evaluated the performance using various evaluation measures to assess the effectiveness of the DL-PPI framework in predicting PPI. In addition, the performance of the proposed method was compared with the methods previously reported in the literature. Finally, a generalizability analysis is carried out to explore how well the model performs on unseen data and conduct ablation experiments to understand the contribution of individual component to the overall performance.

The evaluation of the method involves measuring its accuracy, precision, recall, F1 score, and other relevant metrics on the three datasets. The paper analyzes the results to assess the model’s ability to predict PPI and identify the most informative features contributing to accurate predictions.

Overall, the Results section provides a comprehensive evaluation of the proposed DL-PPI framework, its comparison with existing methods, and a detailed analysis of its generalizability and key features. These findings contribute to a better understanding of the model’s capabilities and potential implications for future research and practical applications in the domain of Protein–Protein Interaction prediction.

### Baselines

In the experimental setup, the paper utilized four different datasets: SHS27k, SHS148k, STRING and Yeast datasets as the basis for comparison with four state-of-the-art protein interaction prediction models: DNN-PPI [59], PIPR [52], TAGPPI [60] and GNN-PPI [43]. All the models were trained and evaluated using these datasets. GNN-PPI is the primary benchmark model.

### Evaluation criteria

In the evaluation of the proposed DL-PPI method, the primary criterion employed was the Micro-F1 metric. The choice of Micro-F1 is suitable to this problem, as it considers data distribution in situations where imbalanced data is present, and assigns equal importance to each sample.

The evaluation metrics used to compute the Micro-F1 score included Precision, Recall, and F1-score, defined by Eq. 9. Precision represents the accuracy of positive predictions made by the model, Recall measures the model’s ability to correctly identify positive samples, and F1-score combines both Precision and Recall to provide a balanced assessment of the model’s performance.

Micro-F1 is computed from the point of view of the samples, considering each sample as equal and ignoring the category weights between the samples. By calculating the sum of Precision and Recall for all categories (here 7 categories), the Micro-F1 score was computed based on the Precision, Recall, and F1-score values, providing a comprehensive evaluation of the DL-PPI model’s effectiveness in predicting Protein–Protein Interactions and its capability to handle imbalanced data distributions across various datasets.

9 $$\begin{aligned} {\begin{matrix} &{} Recall = \frac{TP}{TP+FN} \\ &{} Precision = \frac{TP}{TP+FP'} \\ &{} F1-score = 2 \times \frac{Recall \times Precision}{Recall + Precision} \end{matrix}} \end{aligned}$$

where TP, FP, and FN represent the numbers of true-positive, false-positive, and false-negative samples, respectively.

### Experimental settings

In the experimental settings, the Breath-First Search (BFS) and Depth-First Search (DFS) methods proposed in a previous paper [43] was used to partition the dataset for testing purposes. Specifically, 20% of the PPIs were set aside for testing, and the dataset was segmented using three different methods: Random, BFS, and DFS.

Regarding protein features, the authors utilized amino acid sequences [52] and represented each amino acid using an embedding method. This allowed them to effectively capture the multidimensional information of proteins.

For optimization during training, the authors opted for the Adam algorithm [61], a widely used optimization algorithm in deep learning. The model was trained with a fixed maximum length of 2000 amino acids, and only one Graph layer was used for the processing of protein data. The learning rate was set to 0.001.

During the training process, a batch size of 2048 was employed, and the model was trained for 300 epochs. Each epoch represented one complete iteration through the entire training dataset.

These chosen experimental settings ensured a consistent and rigorous evaluation of the DL-PPI model’s performance on the selected datasets. This approach provided a robust basis for comparing the results with other state-of-the-art models and assessing the model’s capabilities in predicting protein–protein interactions.

### Benchmark

In this study, we conduct a comprehensive benchmark analysis to showcase the effectiveness and superiority of the proposed DL-PPI framework by comparing its performance with other state-of-the-art methods commonly used in similar prediction tasks. This comparative evaluation aims to highlight the advancements and advantages of the proposed approach in the field of PPI prediction.

The results of the benchmark analysis are presented in Table 1. It is observed that the DL-PPI model outperforms other methods across different evaluation techniques, establishing itself as the state-of-the-art model in this domain. Additionally, to ensure a fair comparison, we implemented the DNN-PPI model according to the description in their paper since they were unable to obtain the raw materials. The performance of DL-PPI, along with the comparative models, regarding precision and recall metrics, can be referenced in detail within Additional file 1:  Comparison of Precision and Recall. This additional resource provides comprehensive insights into the models’ comparative performance, shedding light on their precision and recall capabilities.

**Table 1:** Performance of DL-PPI on three datasets in relation to comparative methods

Dataset	Partition scheme	Method				
		DNN-PPI	TAGPPI	PIPR	GNN-PPI	DL-PPI
SHS27k	Random	72.06	85.46	84.28	87.35	**89.12**
	BFS	50.26	49.68	47.39	68.67	**72.95**
	DFS	59.43	63.57	54.25	71.82	**78.07**
SHS148k	Random	87.26	89.21	91.04	90.07	**92.49**
	BFS	56.44	55.9	59.87	67.42	**68.87**
	DFS	59.18	67.35	62.66	84.05	**85.45**
STRING	Random	82.04	89.03	92.76	93.61	**94.85**
	BFS	57.89	58.93	57.15	76.85	**77.53**
	DFS	59.52	68.04	65.48	90.38	**92.76**

The numbers in bold indicate the best performance

The results are reported as the Micro-F1 scores

Under the Random assessment, most of the models demonstrate a significant performance advantage over the BFS and DFS methods. This observation suggests that these models exhibit a strong ability to effectively learn protein models with discretely distributed features.

Furthermore, the DL-PPI model stands out as it does not experience a substantial decrease in performance under the BFS and DFS evaluations compared to the other methods. This indicates that the DL-PPI model is adept at learning more informative features from the protein neighbourhood nodes, leading to higher accuracy in predicting novel protein interactions.

**Fig. 4:**
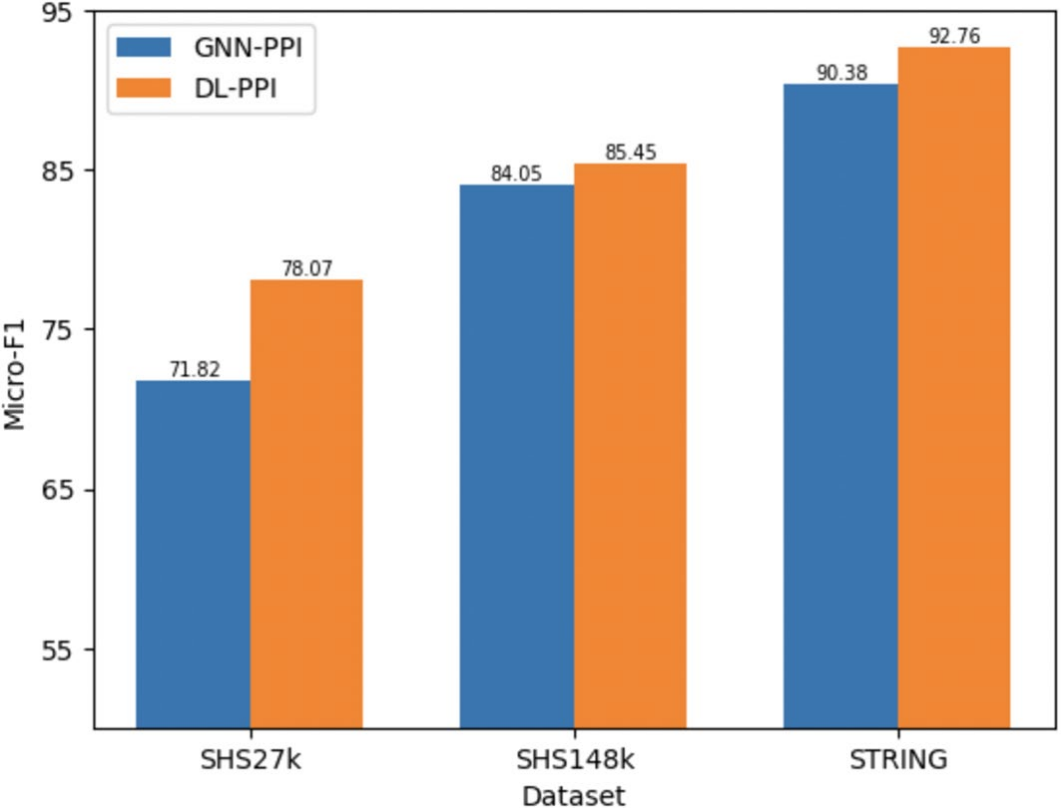
Performance comparison of GNN-PPI (baseline) and DL-PPI

Fig. 4 illustrates the performance comparison between GNN-PPI (baseline) and DL-PPI, conducted using three datasets, and all datasets were evaluated using the DFS test set partitioning method. The evaluation metric utilized was the micro-F1 score for multi-label PPI prediction.

In addition, the evaluation of the method was conducted using the Yeast dataset, and DL-PPI was compared to four other models. All models listed in Table 2 were trained until convergence under a fivefold cross-validation setting. Table 2 reveals that the model under examination outperformed all other models across various evaluation metrics. It excelled in precision, recall, and F1-score, showcasing its significant advantage over the competing models. This noteworthy achievement underscores the model’s robustness and its ability to deliver superior results in the context of the evaluation.

**Table 2:** The performance comparison between DL-PPI and four state-of-the-art sequence-based methods on the Yeast dataset

Methods	Precision (%)	Recall (%)	Micro-F1 (%)
PIPR	97.04	97.9	97.09
TAGPPI	98.1	97.5	97.8
DNN-PPI	95.4	94.84	
GNN-PPI	96.9	96.86	96.76
DL-PPI	97.90	97.93	95.12 97.91

Overall, these findings further validate the effectiveness of the DL-PPI model and highlight its potential for accurately predicting protein–protein interactions, especially in scenarios involving unknown proteins.

### Model generalization

The researchers conducted ablation experiments to systematically remove or modify specific components of the DL-PPI framework. This enabled them to gain valuable insights into the individual contributions and impact of these components on the overall predictive performance.

To assess the model’s generalization ability on an unknown dataset, a comparison experiment was designed, as presented in Table 3. The researchers performed a performance comparison between the test set with homologous proteins (testset-homologous) and the test set with unknown proteins (testset-unknown) under various test set partitioning conditions. The results indicate that the generalization ability of the BFS method for dataset partitioning is relatively more prominent. Furthermore, the comparison demonstrates that the DL-PPI model exhibits superior generalization ability compared to GNN, regardless of the dataset partitioning method used. It highlights the model’s ability to effectively predict protein–protein interactions even when dealing with unseen or unfamiliar protein data, making it a robust and reliable approach for real-world applications.

**Table 3:** Performance comparison between testset-homologous and testset-unknown under various test set partitioning conditions

Method	Trainset	Testset	Random	BFS	DFS
GNN-PPI	SHS27k-Train	SHS27k-Test	87.11	62.10	71.19
		STRING	66.85	66.39	67.43
	SHS148k-Train	SHS148k-Test	91.68	73.88	82.54
		STRING	73.12	67.43	70.64
DL-PPI	SHS27k-Train	SHS27k-Test	**89.29**	**66.63**	**81.13**
		STRING	**68.48**	66.06	**81.01**
	SHS148k-Train	SHS148k-Test	**91.69**	**75.61**	**83.04**
		STRING	66.66	**69.21**	**72.36**

The numbers in bold indicate the best performance

The Micro-F1 evaluation metric is used here

### Ablation experiment

#### Impact of each component

This module were conducted to assess the individual contributions of each component in the DL-PPI model. The experiments were performed on the SHS27k, SHS148k, and STRING datasets using the random classification method with an epoch of 200. Multiple combinations of Inception, Attention, and FRN were examined to determine their impact on the overall performance.

The results of the ablation studies are presented in Table 4. It can be observed that the combined use of Inception, Attention, and FRN yields more effective results compared to using each component independently. This suggests that these components complement each other and synergistically enhance the predictive performance of the DL-PPI model. The ablation experiments validate the validity and significance of integrating these components into the overall framework, further supporting the superiority of the DL-PPI model in protein–protein interaction prediction tasks.

**Table 4:** Study of individual components

Module	SHS27k	SHS148k	STRING
Inception	86.72	88.24	91.36
Attention	88.94	91.65	92.03
FRN	88.06	93.51	93.03
Inception + attention	87.35	90.07	92.82
Inception + FRN	89.02	91.74	92.97
Attention + FRN	88.91	94.14	93.26
Inception + attention + FRN	**89.12**	**92.49**	**93.65**

The numbers in bold indicate the best performance

The Micro-F1 evaluation metric is used here

#### Comparison of NTN and FRN

**Fig. 5:**
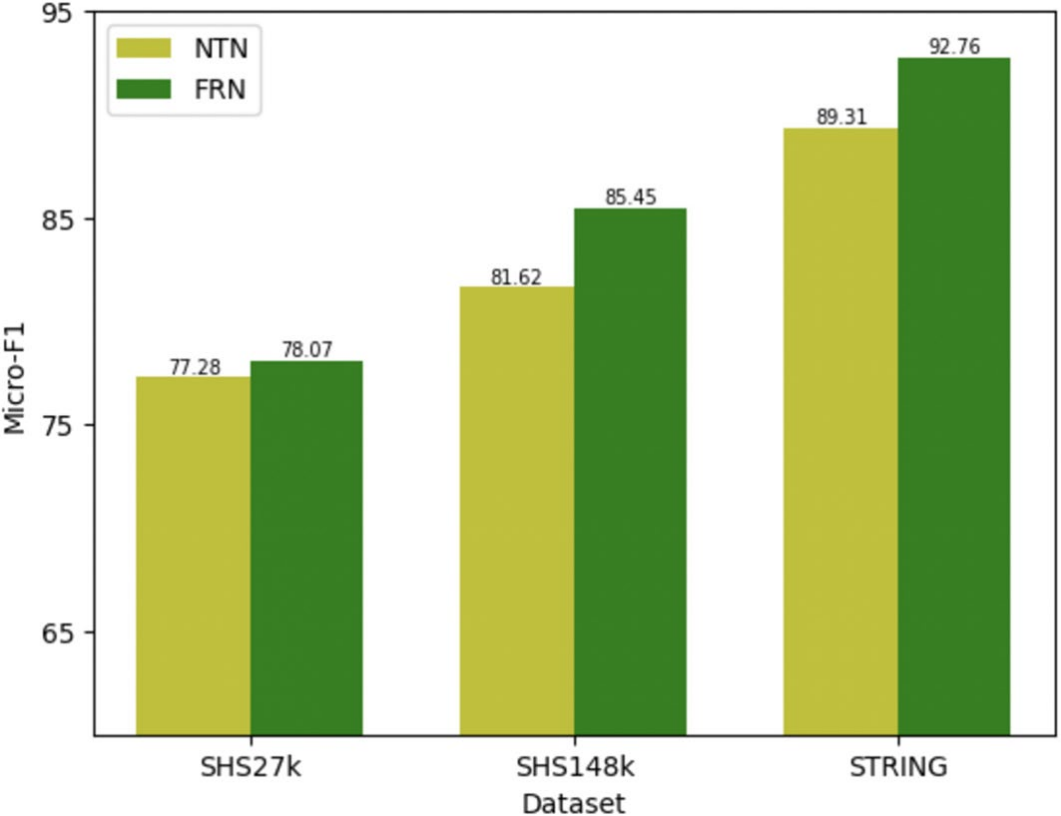
Performance comparison of NTN and FRN

The objective of this experiment is to conduct a comparative analysis between the FRN (Feature Relational Reasoning Network) module in DL-PPI and the method NTN proposed in the paper Neural Tensor Networks [58]. The experiments were conducted on the SHS27k, SHS148k, and STRING datasets using the DFS method for data segmentation, with an epoch of 200. The results, as depicted in Fig. 5, demonstrate that the FRN method integrated within DL-PPI exhibits superior performance compared to the method proposed in the paper NTN across all datasets.

### Enrichment analysis

**Fig. 6:**
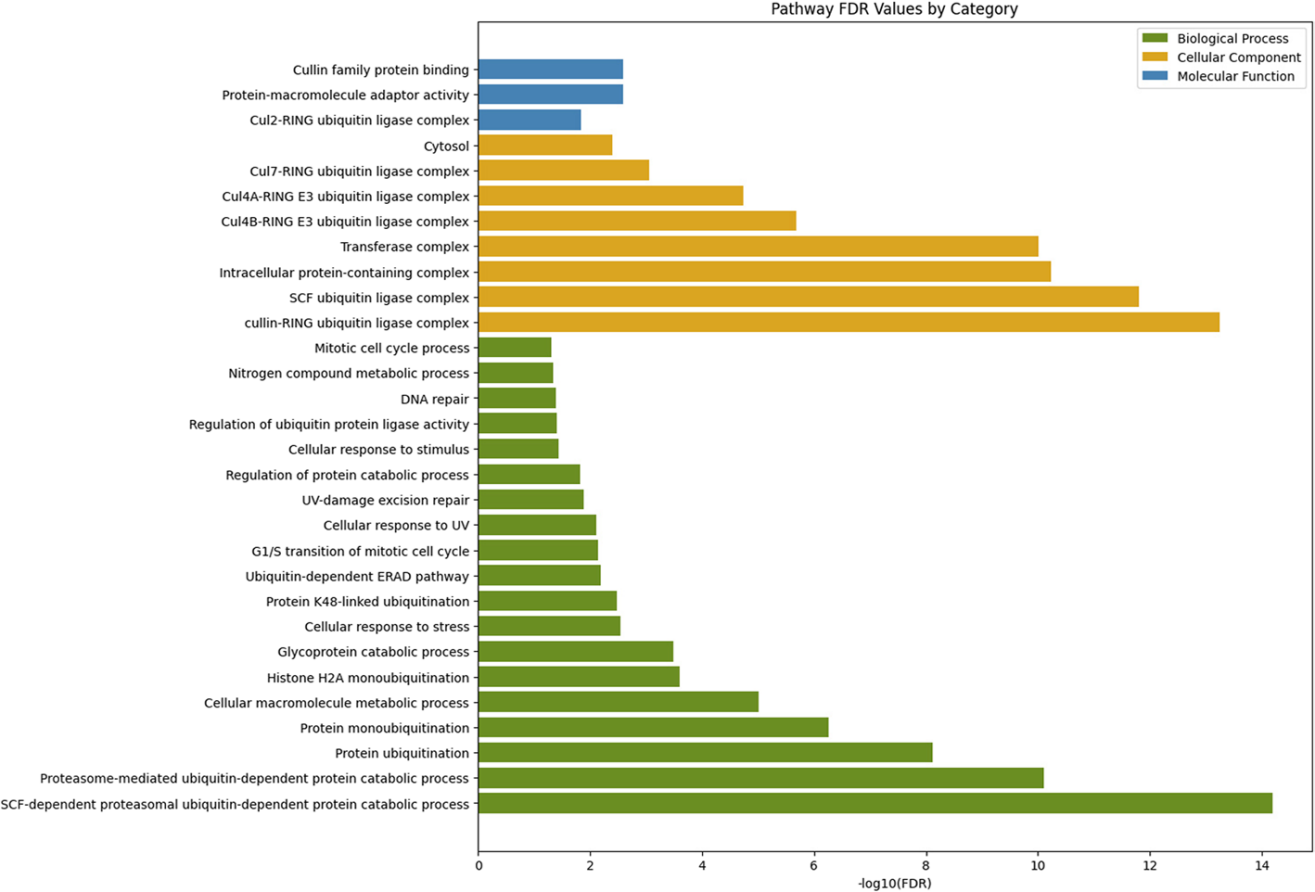
Protein functional analysis pathway diagram—gene ontology

**Fig. 7:**
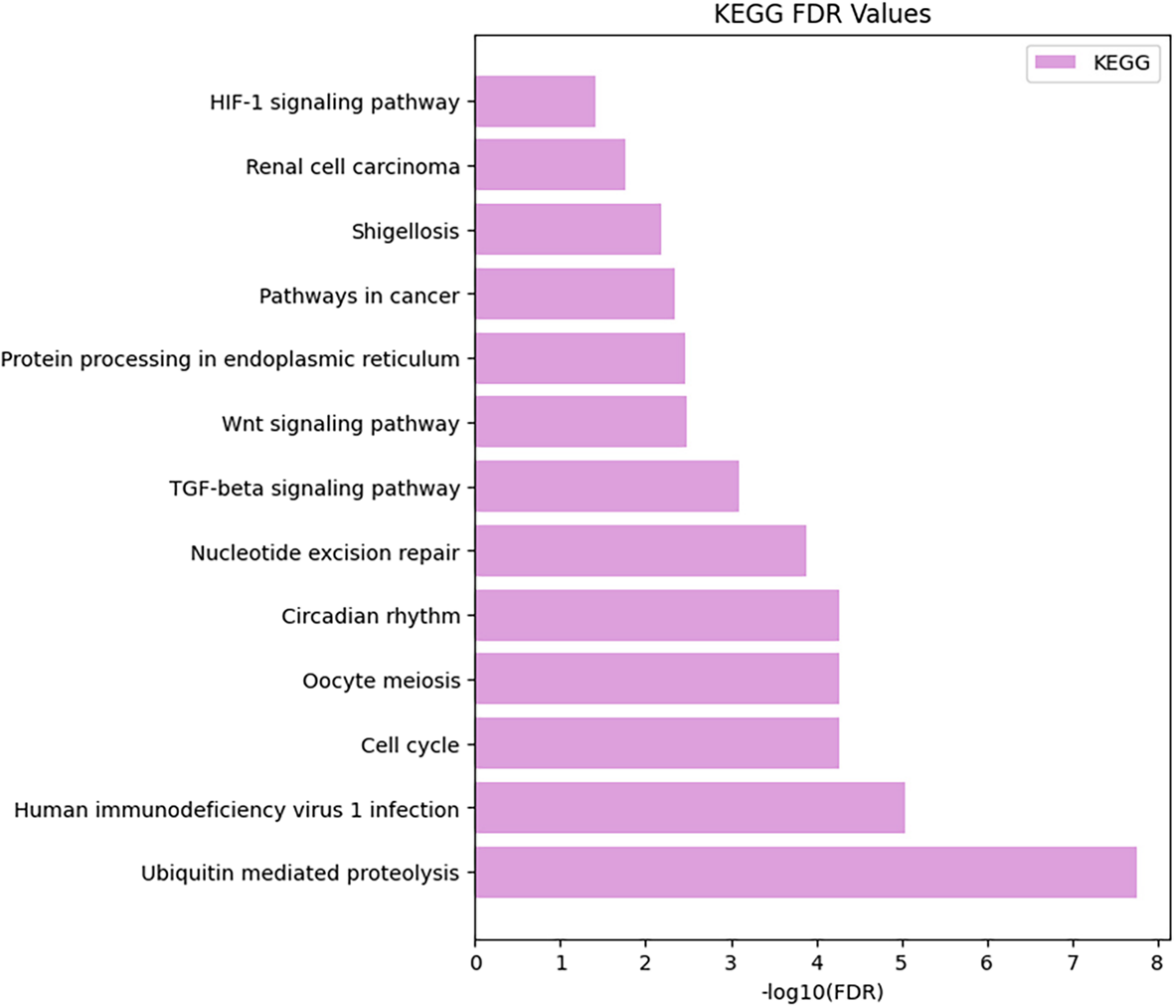
Protein functional analysis pathway diagram—KEGG

In this section, we enhance the comprehensiveness and persuasiveness of our experiments through horizontal validation. Subsequently, Gene Ontology (GO) enrichment analysis and Kyoto Encyclopedia of Genes and Genomes (KEGG) enrichment analysis were conducted on the predicted proteins within the STRING database, as illustrated in Figs. 6 and 7, respectively.

GO is a database created by the GeneOntology Consortium to establish a standard semantic vocabulary for qualifying and describing the functions of genes and proteins across a wide range of species. GO is one of several biological ontology languages that provide a systematic way of defining a three-layer structure for describing the functions of gene products. They divide the function of a gene into three parts: Cellular Component (CC), Molecular Function (MF), and Biological Process (BP).

KEGG was established in 1995 as a database integrating genomic, chemical, and systemic-functional information. KEGG combines gene catalogs derived from genomes that have been completely sequenced with higher level cellular, species, and ecosystem-level system function associations.

Horizontal coordinate suggests the three basic GO categories (Biological Process, Cellular Component, Molecular Function) and the next level of TERMs for each category, from which you can see which specific TERMs describe BP, CC or MF. The vertical coordinate indicates the number of genes annotated to a term (the term and its subterms). The results in Fig. 6 show that the molecular functions of these proteins are predominantly associated with the Cullin family proteins binding and the Protein-macromolecule adaptor activity.

The results in Fig. 7 show that, Ubiquitin mediated proteolysis has the highest number of entries under which enrichment is relatively high.

## Conclusion

In conclusion, this paper have introduced DL-PPI, a novel deep learning-based model for protein–protein interaction prediction that solely relies on protein sequences as input data. Unlike existing methods with limited generalization ability for unknown proteins, DL-PPI leverages a more profound understanding of protein feature interactions to predict interactions involving novel proteins. The model involves preprocessing of protein data, node feature extraction, graph level feature extraction, attention module, and FRN module, culminating in prediction and classification. Through extensive experiments, we have demonstrated the effectiveness of the Attention and FRN modules in improving the model’s performance.

The experimental results have shown that DL-PPI surpasses state-of-the-art PPI prediction methods, particularly in predicting interactions with previously unseen proteins. The ability to accurately predict novel protein–protein interactions makes DL-PPI a valuable tool for advancing the field of protein interaction prediction and opens up possibilities for various applications in the life sciences.

### Supplementary Information

**Additional file 1:**  Comparison of Precision and Recall.

## Data Availability

The STRING Dataset are available at https://cn.string-db.org. All datasets in this study are available at https://github.com/WuBoFu/DL-PPI.git.
