# Supplementary material for: DL-PPI: a method on prediction of sequenced protein–protein interaction based on deep learning
Source: BMC Bioinformatics. 2023 Dec 14;24:473. doi: 10.1186/s12859-023-05594-5 (PMC10722729; doi:10.1186/s12859-023-05594-5)
Supplement: Supplementary file 1 — Additional file 1: Comparison of Precision and Recall. [file 12859_2023_5594_MOESM1_ESM.pdf]

Appendix A Comparison of Precision and Recall

Table A1 Precision of DL-PPI and comparative methods on three datasets.

Dataset	Partition Scheme	Precision(%)				
		DNN-PPI	TAGPPI	PIPR	GNN-PPI	DL-PPI
SHS27k	Random	70.45	86.33	79.92	90.08	90.71
	BFS	68.92	68.16	39.7	70.78	70.41
	DFS	68.99	69.81	68.56	72.27	79.3
SHS148k	Random	88.91	89.85	91.86	91.15	**9**2.9
	BFS	67.42	67.98	72.49	70.78	70.39
	DFS	76.77	70.69	71.94	87.23	86.75
STRING	Random	84.97	90.05	92.79	94.42	95.28
	BFS	61.28	71.06	75.69	77.53	78.88
	DFS	67.16	77.82	69.81	91.02	93.47

Table A2 Recall of DL-PPI and comparative methods on three datasets.

Dataset	Partition Scheme	Recall(%)				
		DNN-PPI	TAGPPI	PIPR	GNN-PPI	DL-PPI
SHS27k	Random	75.46	84.85	89.46	84.3	88.45
	BFS	39.57	39.44	55.39	63.94	72.17
	DFS	52.57	62.37	45.34	72.87	73.45
SHS148k	Random	85.45	88.72	90.07	89.16	92.15
	BFS	48.81	55.23	52.57	63.94	68.68
	DFS	48.36	65.85	56.4	81.88	85.49
STRING	Random	79.3	87.99	91.13	92.86	94.22
	BFS	51.49	50.5	45.86	75.5	75.31
	DFS	53.3	60.78	62.37	89.31	91.99
